# Neonatal sepsis due to Coxsackievirus B3 complicated by liver failure and pulmonary hemorrhage

**DOI:** 10.1515/crpm-2021-0085

**Published:** 2022-03-04

**Authors:** Rasmey Thach, Lorenzo Gitto

**Affiliations:** Department of Medicine, William Beaumont Army Medical Center, El paso, TX, USA; Department of Pathology, State University of New York Upstate Medical University, Syracuse, USA

**Keywords:** autopsy, Coxsackievirus B3, enterovirus, hemorrhage, multiorgan failure, neonatal sepsis

## Abstract

**Objectives:**

Coxsackievirus B3 (CVB3) is a single-stranded RNA included in the “Human Enterovirus B” category associated with multiple, even severe, health issues in humans. Newborns are at risk of life-threatening conditions due to enteroviral infections. In newborns, the infection can be transmitted vertically, intrapartum or postpartum, and potentially through breast milk. Neonatal sepsis may result in severe complications, such as liver failure and pulmonary hemorrhage, with subsequent death.

**Case presentation:**

A male newborn was admitted to the emergency department with fever, generalized hypotonia, hypo-reactivity to external stimuli, multiple episodes of apnea and desaturation, and metabolic acidosis. Laboratory studies revealed disseminated intravascular coagulation, and evidence of progressive multiorgan failure. Polymerase chain reaction performed on specimens collected at the time of admission returned positive for Enterovirus, specifically Coxsackievirus B3 VP1 gene. The patient eventually succumbed after several days due to severe sepsis, despite aggressive treatment with immunoglobulins and Pleconaril. An autopsy revealed hemorrhage in the lung, liver, heart, and gastric mucosa.

**Conclusions:**

Enteroviral neonatal infections should be included in the differential diagnosis of a newborn presenting with fever, failure to thrive, and hyporeactivity, especially if symptoms arise during the classic CVB3 season. Maternal medical history should be reviewed for any possible febrile symptoms associated with a recent enterovirus infection. Aggressive treatment with immunoglobulins and, if available, Pleconaril could effectively treat the infection.

## Introduction

Coxsackieviruses are nonenveloped viruses with linear single-stranded RNA, included in the Enterovirus group, belonging to the “Picornaviridae” family. Coxsackievirus B3 (CVB3) is included in the “Human Enterovirus B” category [1], and it is associated with multiple severe health issues in humans, such as myocarditis, meningitis, and pancreatitis [2], [3], [4], Coxsackievirus infections exhibit a seasonal variation pattern with peak infectivity between June and October. Most enterovirus infections are self-limited, and no specific therapy is needed. However, in newborns, enteroviral infections are potentially life-threatening due to their immature immune systems. We report a death due to CVB3 neonatal sepsis associated with multiorgan failure.

## Case presentation

A male newborn (35w + 5) presented to the emergency department on his sixth day of life for progressive failure to thrive associated with a 100-g weight loss. The mother reported having a fever, cough, and sore throat the day prior to delivery. At birth, the Apgar Scores were 9, 9, and 10 at 1, 5, and 10 min, respectively. Other than a late preterm delivery, there were no other abnormalities detected. The mother and newborn were discharged with a plan to return for follow-up in three days. At follow-up, the newborn was in good condition but subsequently developed a failure to thrive in the following days.

On admission, the baby was mildly febrile to 37.5 °C and he exhibited generalized hypotonia with hypo-reactivity to external stimuli. The patient had multiple apneic episodes resulting in oxygen desaturations; an arterial blood gas drawn revealed a pH of 7.21, consistent with metabolic acidosis. As neither viral nor active bacterial infections could be excluded, broad-spectrum antibiotics were initiated, and blood cultures were obtained. A markedly saturation to 75% persisted, requiring transfer to the pediatric intensive care unit where the patient was placed into a neonatal incubator with escalation of oxygen therapy. Laboratory studies revealed disseminated intravascular coagulation (DIC), and fresh frozen plasma and platelets were transfused. The patient also received vitamin K and continued with broad-spectrum antibiotics. The following day, the patient developed diffuse generalized jaundice and became oliguric with labs revealing hepatocellular injury (elevation in aspartate aminotransferase [AST] and alanine aminotransferase [ALT]), and thrombocytopenia to <15,000. Despite aggressive treatment, the metabolic acidosis persisted. Polymerase chain reaction returned positive for enterovirus, specifically *CVB3 VP1* gene, on the third day.

There was a progressive deterioration of hepatic and renal function in the following days despite antibiotics, immunoglobulin therapy, and intensive care level support. Pleconaril, an antiviral drug from a capsid inhibitor class, was also added to the therapeutic regimen. The baby developed tonic-clonic contractions at the upper and lower extremities and massive pulmonary, hepatic, and gastric bleeding. Four days into the hospital course, the patient experienced a cardiac arrest from which there was no return of spontaneous circulation despite resuscitative measures. Ultimately, the cause of death was sepsis due to Coxsackievirus B3 infection associated with multiorgan failure.

**Figure 1:**
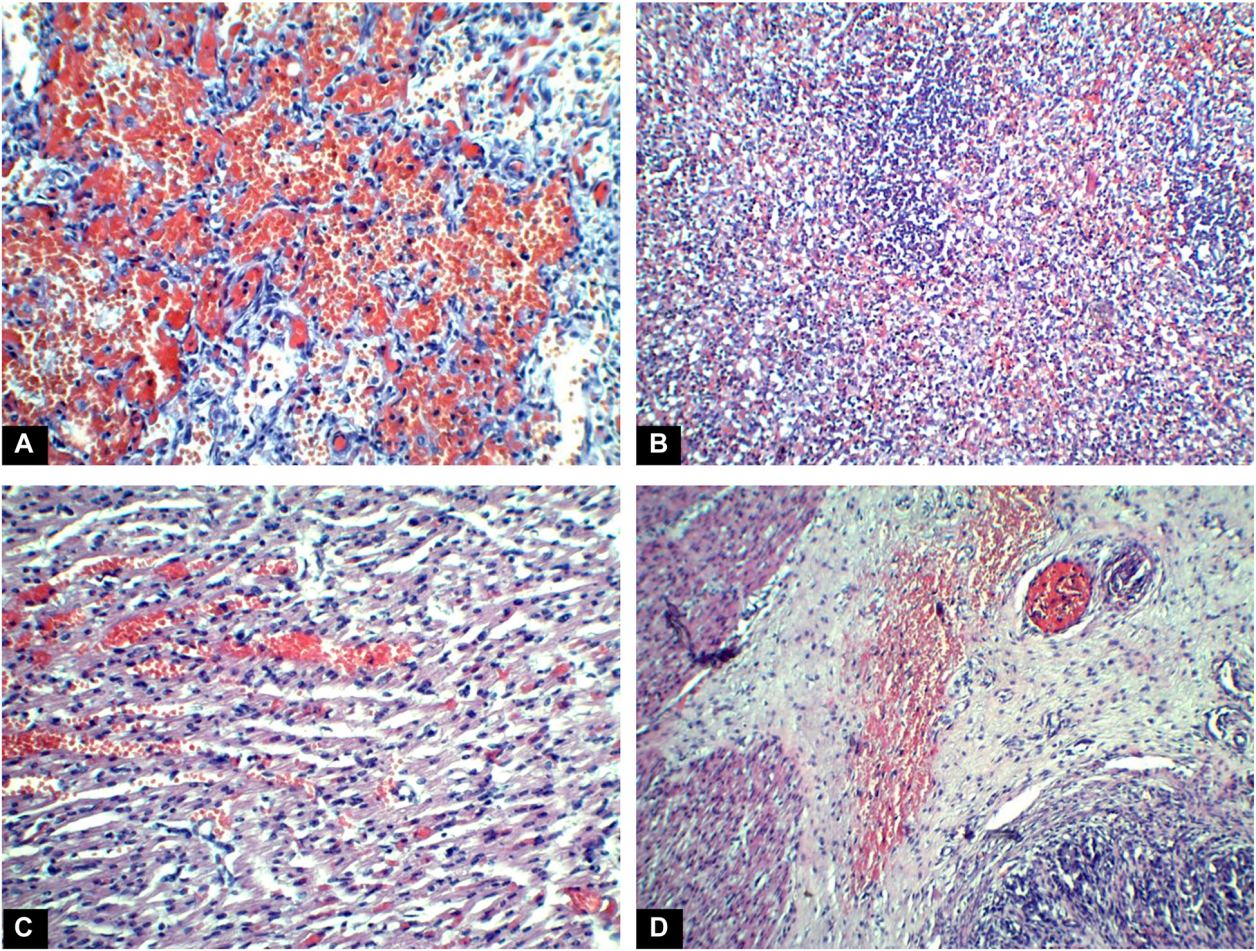
Histology results. (A) Lung. Diffuse intraalveolar hemorrhage (H&E, 200×). (B) Liver. Complete architecture distortion with hepatocytes necrosis and intraparenchymal hemorrhage (H&E, 100×). (C) Heart. Focal interstitial hemorrhage (H&E, 200×). (D) Stomach. Focal submucosal hemorrhage (H&E, 200×).

An autopsy was requested. The external examination did not show any abnormalities. The autopsy revealed multiple hemorrhagic areas in the lungs and liver, subepicardial and kidney petechiae, and gastric hemorrhage. Histology confirmed diffuse lung interalveolar hemorrhage, intraparenchymal liver hemorrhage, heart interstitial hemorrhage, and gastric mucosal focal hemorrhage (Figure 1).

## Discussion

Newborns have an increased risk of infection due to their immature immune system. During gestation, fetal development occurs in a sterile environment which limits the immune system’s stimulation by way of its limited exposure to antigens. Therefore, after birth, newborns exhibit a limited response to external infectious stimuli.

CVB3 primarily binds a specific receptor (coxsackievirus and adenovirus receptor [CAR]), and a co-receptor on the host cell (decay-accelerating factor – DAF or CD55) [5]. After binding the receptors, the virus enters the host cell’s cytoplasm, where it utilizes the host cell’s machinery for its own benefit. Inactive viral proteins and negative-strand RNA intermediates are synthesized. Finally, multiple positive-strand RNA molecules are produced, and the inactive viral proteins get cleaved, thereby activated, resulting in the formation of complete virions [6].

CVB3 are transmissible through many routes. Commonly, transmission occurs through fecal-oral or respiratory mechanisms [7]. In newborns, the infection can be transmitted vertically (transmission directly from the mother to the embryo, fetus, or baby during pregnancy) [8], intrapartum (exposure to infected maternal blood and/or genital secretions during delivery) [9], or postpartum (after delivery). There are also case reports of transmission through breast milk [10].

CVB3 and other Enteroviruses commonly cause infection in newborns and children [11]. A severe, life-threatening complication of newborn infection by CVB3 is sepsis, which is usually defined as a systemic infection occurring in infants at ≤28 days [12]. Neonatal sepsis is a significant cause of morbidity and mortality of newborns. Among neonatal sepsis complications, disseminated intravascular coagulation is one of the major causes of hemodynamic instability in newborns. During sepsis, the responsible pathogen causes the release of proinflammatory cytokines and activation of monocytes, resulting in the up-regulation of tissue factors, which eventually activates the coagulation cascade. Moreover, cytokines stimulate the endothelial cells to produce plasminogen activator inhibitors, thus decreasing fibrinolysis [13].

The hepatic coagulation system is still immature in newborns, and liver damage results in more severe coagulation abnormalities [14]. Because of progressive liver failure, DIC develops and leads to uncontrollable hemorrhagic diastasis and eventually to multiorgan failure.

In the present case, an RT-PCR assay was positive for the *CVB3 VP1* gene. This gene encodes for the viral capsid protein VP1 that induces cell cycle arrest at the G1 phase and upregulates specific proteins (such as the Heat Shock Protein 70 [HSP70]), increasing viral replication ability and facilitating CVB3 infection [15]. The infection rapidly evolved into sepsis, leading to progressive liver failure, a life-threatening complication in newborns with enteroviruses infection [16]. Laboratory evidence of hepatocyte necrosis (elevation in ALT and AST) confirmed the progressive hepatic involvement. This finding was also confirmed at the postmortem histology examination of the liver that revealed diffuse hepatocytes necrosis and intraparenchymal hemorrhage. The progressive liver failure led to DIC with uncontrollable severe multiorgan bleeding.

Pulmonary hemorrhage in CVB3 infection is a rare complication characterized by high mortality in the neonatal population [17]. It is associated with a rapid, progressive respiratory failure with hemoptysis, hypoxemia, bradycardia, and hypotension. It is thought that pulmonary hemorrhage is a consequence of an increased pulmonary blood flow due to a possible patent ductus arteriosus. In the present case, the pulmonary hemorrhage was detected the day before the patient expired, suggesting an irreversible terminal evolution of the systemic critical illness. Histological examination revealed diffuse intraalveolar hemorrhage in all lung samples collected during the autopsy, lending evidence to the severity of the decedent’s respiratory status prior to death.

Additional findings, such as gastric hemorrhage and myocardial hemorrhage, further provided evidence for the terminal multiorgan failure secondary to the hemorrhagic diastasis in the setting of DIC.

There are several clinical symptoms that should raise suspicion for possible enteroviral infection which include fever, failure to thrive, hyporeactivity, irritability, seizure. However, these clinical symptoms comprise a presentation that is common in many neonatal infections. Thus, it is critical to investigate the maternal medical history in the period prior to delivery. It has been shown that symptoms such as fever and cough are common in mothers who are infected with Enterovirus [18], thus facilitating the clinician’s suspicion of a neonatal infection sustained by the same organism.

There is no specific therapy for CVB3 infections, and the treatment is largely supportive care. In the presented case, the newborn’s escalating oxygen requirement necessitated appropriate respiratory support from conventional oxygen supplementation to orotracheal intubation. Hemodynamically, the patient required vasopressors and multiple red blood cell transfusions. Platelet and fresh frozen plasma were also transfused. Empiric broad-spectrum antibiotics, furosemide, phenobarbital, vitamin K, and carglumic acid were administered to treat a possible bacterial infection, anuria, seizure, and liver disease, respectively. Finally, immunoglobulins and Pleconaril were also administered. Immunoglobulins are considered an essential therapy to produce serum neutralizing antibodies to the virus and protect against cardiac damage in Coxsackievirus B infections [19]. Pleconaril is an antiviral drug from a capsid inhibitor class that prevents the virus from attaching to cellular receptors by integrating within a hydrophobic pocket inside the virion, leading to rigidification and compression of the viral capsid. Although its efficacy is still under investigation, recent research suggests that Pleconaril might become potent, selective anti-coxsackievirus inhibitors with specific activity against the CVB3 [20]. Its use is considered reasonable due to the high mortality of neonatal infection.

## Conclusions

Enterovirus neonatal infections should be considered in the differential diagnosis of a newborn presenting with fever, failure to thrive, and hyporeactivity. Symptom presentation during the classic CVB3 season may help raise the clinical suspicion. Maternal medical history should be reviewed for any possible fever associated with a recent enterovirus infection. Once there is a clinical suspicion of Enterovirus, rapid RT-PCR should be utilized promptly to evaluate for the presence of the viral genome to facilitate a timely diagnosis and appropriate treatment. As most infections in preterm infants, serious complications are expected, and death usually results from a multiorgan failure without specific findings. However, pulmonary hemorrhage is an uncommon, often unrecognized, but extremely dangerous complication of enterovirus infection in preterm infants. It should be especially suspected in premature newborns. It is critical to suspect and anticipate this uncommon complication that usually represents the terminal event leading to death. Since pulmonary hemorrhage is associated with a poor prognosis, its early, aggressive management may lead to a less severe outcome. Supportive therapy, aggressive treatment with immunoglobulins and, if available, Pleconaril could effectively treat the infection. In cases of fatal outcomes, an autopsy should be offered to the family to confirm the extent of the disease and detect findings related to the multiorgan involvement.
